# Cardiovascular disease risk prediction in multi-ethnic Asian populations: evidence from two population-based cohorts in Singapore

**DOI:** 10.1016/j.lanwpc.2025.101794

**Published:** 2026-01-02

**Authors:** Charlie G.Y. Lim, Crystal C.Y. Chong, Yvonne H.M. Wong, Jiali Yao, Stefen Ma, John C. Chambers, Khung Keong Yeo, E Shyong Tai, Jasper Tromp, Rob M. van Dam, Saima Hilal, Charumathi Sabanayagam, Ching-Yu Cheng, Xueling Sim

**Affiliations:** a Saw Swee Hock School of Public Health, National University of Singapore and National University Health System, 12 Science Drive 2, 117549, Singapore; b Singapore Eye Research Institute, Singapore National Eye Centre, 20 College Road Discovery Tower Level 6, 169856, Singapore; c Ministry of Health, Singapore, 16 College Road College of Medicine Building, 169854, Singapore; d Nanyang Technological University Lee Kong Chian School of Medicine, Level 18 Clinical Sciences Building, 11 Mandalay Road, 308232, Singapore; e Department of Epidemiology and Biostatistics, School of Public Health, Imperial College London, 152 Medical School, St Mary's Campus, London, W2 1NY, UK; f National Heart Centre Singapore, 5 Hospital Dr, 169609, Singapore; g Duke-National University of Singapore Medical School, 8 College Rd, 169857, Singapore; h Department of Medicine, Yong Loo Lin School of Medicine, National University of Singapore, 10 Medical Dr, 117597, Singapore; i Department of Cardiology, University Medical Centre Groningen, University of Groningen, the Netherlands; j Departments of Exercise and Nutrition Sciences and Epidemiology, Milken Institute School of Public Health, The George Washington University, 950 New Hampshire Ave, NW, Washington, DC 20052, USA; k Department of Pharmacology, Yong Loo Lin School of Medicine, National University of Singapore, Singapore, 16 Medical Drive, 117600, Singapore; l Department of Ophthalmology, Yong Loo Lin School of Medicine, National University of Singapore, 1E Kent Ridge Road, 119228, Singapore

**Keywords:** Cardiovascular disease, Risk assessment, Risk prediction, Asia, Framingham risk score, Pooled cohort equations, SCORE2

## Abstract

**Background:**

The rising burden of cardiovascular diseases (CVD) in Asia requires risk assessment tools tailored to Asian populations. Therefore, we recalibrated the ACC/AHA Pooled Cohort Equations for non-Hispanic Whites (PCE-W) and compared its performance in predicting 10-year CVD risk with two other established CVD prediction models that have been recently recalibrated for Asian populations.

**Methods:**

We used data from the Singapore Multi-Ethnic Cohort (MEC1) and the Singapore Epidemiology of Eye Diseases (SEED) cohort comprising ethnic Chinese, Indian, and Malay participants. The PCE-W was recalibrated using data from MEC1, externally validated in the SEED cohort, and compared against the Singapore-modified Framingham Risk Score (SG-FRS-2023) and the SCORE2 Asia–Pacific model using the concordance index (C-index). Calibration was assessed using the calibration-in-the-large method, the calibration slope, and a goodness-of-fit test.

**Findings:**

All three models demonstrated possibly helpful to clearly useful discrimination in MEC1 and SEED, with overall C-indices ranging from 0.728 to 0.811. The recalibrated PCE-W outperformed the original PCE-W in MEC1 and SEED, although some misestimations remained among Chinese men and women and Malay women (calibration-in-the-large ranged from −0.479 to 0.260). The SG-FRS-2023 displayed generally satisfactory calibration across both MEC1 and SEED but tended to overestimate risk in Chinese (calibration-in-the-large −0.671) and Indian men (calibration-in-the-large −0.214) in the SEED cohort. The SCORE2 Asia–Pacific model performed satisfactorily among Indians but overestimated risk in Chinese (calibration-in-the-large ranged from −0.570 to −1.185) and showed poor model fit in Malays.

**Interpretation:**

The recalibrated PCE-W, SG-FRS-2023, and SCORE2 Asia–Pacific model demonstrated possibly helpful to clearly useful discrimination across two multi-ethnic cohorts in Singapore. In terms of calibration, the recalibrated PCE-W and SG-FRS-2023, both recalibrated using local data, performed better than the SCORE2 Asia–Pacific model. Our study supports the use of the established CVD prediction models in Asian populations following appropriate local recalibration.

**Funding:**

This work was supported by the Singapore Ministry of Health’s National Medical Research Council and the Singapore 10.13039/501100012415Biomedical Research Council.

**Research in context**

**Evidence before this study**

The incidence of cardiovascular disease (CVD) mortality is projected to double by 2050 in Asia and signals an urgent need for CVD risk assessment tools tailored for Asian populations. We searched PubMed without language restrictions for relevant studies on CVD risk prediction in Asia, using search terms (“CVD” OR “cardiovascular” OR “cardiovascular disease”) AND (“prediction” OR “risk assessment”) AND (“Asia” OR “Asia Pacific”). Most Asian populations are using CVD risk assessment tools developed in Western populations, with the Framingham Risk Score and its variations being the most commonly used models. The Pooled Cohort Equations (PCE), introduced to include stroke outcomes that were not included in Framingham Risk Score, was found to misestimate risk in several Asian populations. Most recently, the European Systematic COronary Risk Evaluation 2 (SCORE2) model was recalibrated to different risk regions for use in the Asia–Pacific region. While the SCORE2 Asia–Pacific model demonstrated satisfactory calibration in several Asian cohorts, its performance across different ethnic groups was not evaluated. This indicates a need to evaluate and recalibrate these emerging risk models before use in Asian populations.

**Added value of this study**

Using data from two independent multi-ethnic cohorts in Singapore comprising ethnic Chinese, Malay, and Indian participants, we recalibrated the PCE and compared its performance with the Singapore-modified Framingham Risk Score (SG-FRS-2023) and the SCORE2 Asia–Pacific model. We show that (i) all three models demonstrated possibly helpful to clearly useful discrimination across sex and ethnicity across both of our multi-ethnic cohorts, and (ii) the SG-FRS-2023 and recalibrated PCE demonstrated better calibration than the SCORE2 Asia–Pacific model in our Singapore cohorts. Our study is the first to recalibrate the PCE using data from a multi-ethnic Asian cohort with validation in an independent cohort. We included data from ethnic Indians (South Asian) and Malays (Southeast Asian) which are populations that are underrepresented in CVD prediction research.

**Implications of all the available evidence**

All three established CVD prediction models generally demonstrate possibly helpful to clearly useful discrimination (C-statistics >0.60) in Asian populations with diverse ethnic compositions, and our study supports the use of these models in multi-ethnic Asian populations after appropriate recalibration. As atherosclerotic CVD is the leading cause of CVD mortality in Singapore, the recalibrated PCE provides an alternative over SG-FRS-2023 for CVD risk assessment in Singapore.

## Introduction

The incidence of cardiovascular disease (CVD) mortality is projected to double by 2050 in Asia, with atherosclerotic diseases such as ischaemic heart diseases and stroke being the main drivers of the CVD burden.^1^ Lifestyle changes and pharmacotherapy can reduce the risk of CVDs, especially in higher-risk individuals.^2^ It is therefore important to identify individuals at a higher risk of CVDs to enable preventive measures to reduce the CVD burden.

Most Asian populations are using CVD risk assessment tools developed in Western populations.^3^ In Singapore, the current clinical practice guideline for CVD risk assessment is based on a locally recalibrated version of the Framingham-based Adult Treatment Panel (ATP) III model, hereafter referred to as the Singapore-modified Framingham Risk Score (SG-FRS-2023).^4^ Although the ATP III model is one of the most widely validated models internationally,^5^ it is not without limitations. First, the ATP III model was designed to predict the risk of 10-year coronary heart disease (CHD) and does not include stroke as an endpoint.^6^ Furthermore, the ATP III model considers diabetes as a CHD risk equivalent and did not include individuals with diabetes in the model development.^6^

To overcome these limitations, the American College of Cardiology and the American Heart Association (ACC/AHA) introduced the Pooled Cohort Equations (PCE) for the assessment of 10-year atherosclerotic CVD (ASCVD) risk that included CHD and stroke among individuals with or without diabetes. The PCE was developed in several large, racially and geographically diverse populations in the United States and consisted of two sets of sex-specific equations designed for non-Hispanic Whites (PCE-W) or African Americans (PCE-AA).^7^ However, the PCE-W was found to misestimate risk in several Asian cohorts from Korean, Chinese, Southeast Asian, and South Asian populations,^8^, ^9^, ^10^, ^11^, ^12^, ^13^ indicating a need for recalibration before use in these populations.

Recently, the Systematic COronary Risk Evaluation 2 (SCORE2) model was recalibrated using country-specific CVD mortality rates reported by the World Health Organization's Global Health Estimates and region-specific multipliers derived from five countries representing low, moderate, high, and very-high-risk regions in the Asia-Pacific.^14^ The SCORE2 model was originally developed using data from 13 European countries and is recommended by the European Society of Cardiology for CVD risk assessment in Europe.^15^ While the SCORE2 Asia–Pacific model demonstrated satisfactory calibration in several Asian populations including a multi-ethnic Singapore cohort (a low-risk region), its performance across different ethnic groups was not evaluated. This knowledge gap is important given the well-documented differences in CVD risk among different Asian ethnic groups.^16^^,^^17^

Therefore, we aimed to (i) evaluate and recalibrate the PCE-W for use in multi-ethnic Asian populations, and (ii) compare the performance of recalibrated PCE-W, SG-FRS-2023, and SCORE2 Asia–Pacific model in two population-based cohorts in Singapore comprising Chinese (East Asians), Malays (Southeast Asians), and Indians (South Asians).

## Methods

### Study populations

**Fig. 1:**
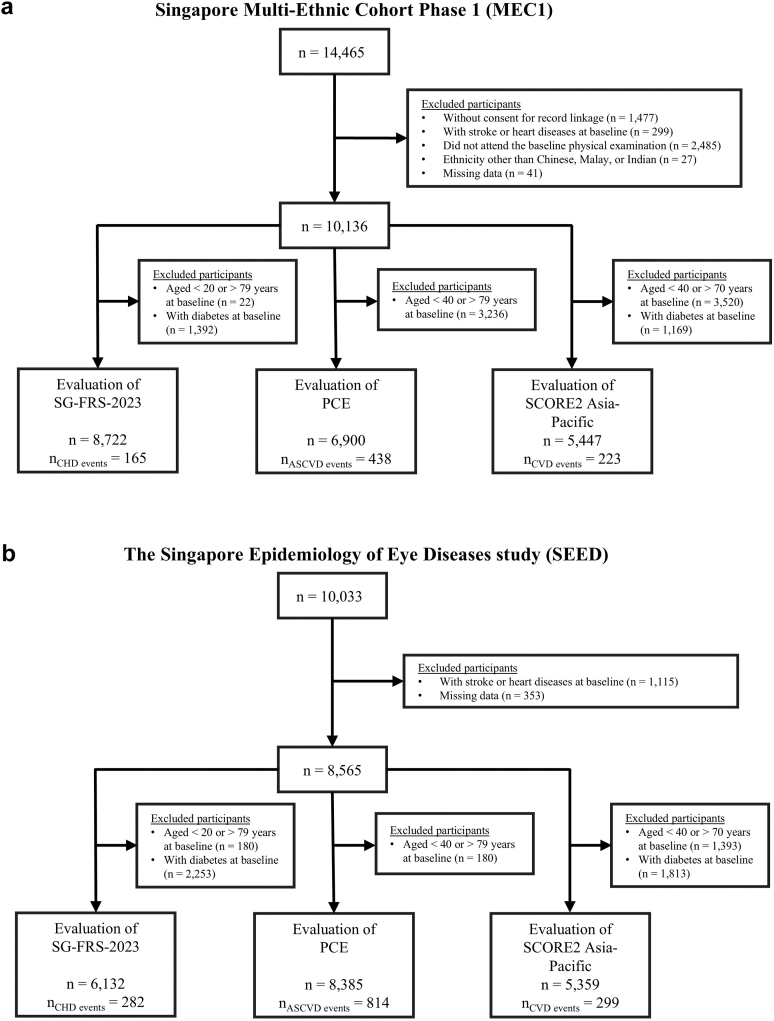
Flow charts detailing inclusion criteria, baseline sample size and incident CVD events for (a) the Singapore Multi-Ethnic Cohort Phase 1 (MEC1) and (b) the Singapore Epidemiology of Eye Diseases study (SEED) for three CVD models (SG-FRS-2023, Pooled Cohort Equations (PCE) and SCORE2 Asia–Pacific).

Data from the Singapore Multi-Ethnic Cohort Phase 1 (MEC1) and the Singapore Epidemiology of Eye Diseases (SEED) cohort were used to evaluate the SG-FRS-2023, PCE-W, and SCORE2 Asia–Pacific model. Details of the MEC1 and SEED cohorts have been described previously.^18^^,^^19^ Briefly, MEC1 is part of a population-based cohort study examining how genetics, lifestyle, and environmental factors impact disease risk. Between 2004 and 2010, 14,465 male and female adult Singapore residents were recruited to the study, with disproportionate sampling stratified by ethnicity to ensure a good representation of the three major ethnic groups in Singapore (Chinese, Malay, and Indian). The SEED cohort comprises 10,033 Singaporean male and female adults recruited between 2004 and 2011 through an age-stratified random sampling of individuals aged 40–80 years old from 15 residential districts in Singapore.^19^ The data and CVD ascertainment flow chart are shown in Fig. 1. We excluded participants without consent for medical record linkage, participants with stroke or heart disease at baseline, participants with ethnicity other than Chinese, Malay, or Indian, and participants with missing data. We further excluded participants based on age and diabetes status, as specified in the exclusion criteria for each prediction model. The final number of participants for model evaluation was as follows: SG-FRS-2023 (8722 for MEC1 and 6132 for SEED), PCE (6900 for MEC1 and 8385 for SEED), and SCORE2 Asia–Pacific (5447 for MEC1 and 5359 for SEED).

### Baseline risk factors

Age, self-reported sex, self-reported ethnicity determined from each participant's National Registration Identity Card, smoking status, personal medical history, and medication were obtained from interviewer-administered questionnaires at baseline. Systolic blood pressure was measured using automated digital monitors (MEC1: Dinamap Carescape V100; SEED: Dinamap model Pro Series DP110X-RW, 100V2). High-density lipoprotein (HDL) and total cholesterol were measured in fasting serum samples in MEC1 (ADVIA 2400 Chemistry System) and non-fasted serum samples in SEED (Beckman Coulter Unicel DXC 800). A history of diabetes mellitus was defined as having a physician-diagnosed diabetes, or an elevated fasting blood glucose level (≥7 mmol/L), random blood glucose level (≥11 mmol/L), or haemoglobin A1c (HbA1c) concentration (≥6.5%) at baseline.

### Outcome ascertainment

The primary outcomes were the incidence of CHD (for SG-FRS-2023), ASCVD (for PCE), and CVD (for SCORE2 Asia–Pacific). The incidence of CHD was defined as the incidence of non-fatal myocardial infarction and ischaemic heart disease mortality (International Classification of Diseases, Tenth Revision [ICD-10] I20–I25). The incidence of ASCVD was defined as the incidence of non-fatal myocardial infarction, non-fatal stroke, ischaemic heart disease mortality (ICD-10 I20–I25), or cerebrovascular disease mortality (ICD-10 I60–I63, I67–I69). The incidence of CVD was defined according to Hageman et al., which included hypertensive diseases, ischaemic heart diseases, arrhythmias and heart failure, cerebrovascular diseases, and atherosclerosis.^14^ These outcomes were ascertained through record linkage with the Singapore National Registry of Diseases Office (NRDO) until 31 December 2019. NRDO identifies cases of myocardial infarction, stroke, and mortality through hospital inpatient discharge summaries from all public healthcare institutions, medical claims from the Ministry of Health, and the death registry from the Ministry of Home Affairs. The event-free survival duration was calculated from the date of recruitment to the date of the first event, the end of follow-up, or death. Participants who did not experience any CVD event during the follow-up were censored.

### Statistical analysis

#### CVD prediction models and predicted risk

A summary of the SG-FRS-2023, PCE, and SCORE2 Asia–Pacific model is provided in Supplementary Table S1. All three models share a common set of predictors, including age, total and HDL cholesterol, systolic blood pressure, hypertension treatment, and smoking history, with the exception of PCE, which also included diabetes as a predictor.

Details of the SG-FRS-2023 algorithm have been reported elsewhere.^4^ Briefly, the SG-FRS-2023 is a recalibrated version of the Framingham-based ATP III model derived by replacing the 10-year average survival from the Framingham-based ATP III model with the sex- and ethnic-specific 10-year average survival from MEC1.^4^ This SG-FRS-2023 algorithm estimates the predicted 10-year CHD risk among individuals aged 20–79 years without diabetes at baseline.

We used the PCE algorithm to estimate the predicted 10-year ASCVD risk among individuals aged 40–79.^7^ Next, we recalibrated the PCE in MEC1 using a modified version of the method used in SCORE2 recalibration.^14^ Details of the method and the rationale for the modifications to the SCORE2 method are provided in Supplementary Note 1, along with Supplementary Tables S2 and S3. Briefly, this method involves deriving sex- and ethnic-specific rescaling factors by relating the age-specific average expected risk to the age-specific average predicted risk. The average 10-year expected risk in MEC1 was estimated using the Kaplan–Meier estimator. The average 10-year predicted risk was calculated using the coefficients from the PCE and the means of the risk factors in MEC1. The rescaling factors were then used to recalibrate the original predicted risk for a given individual. It should be noted that the recalibration method used for the PCE does not change the discrimination of the model.

Lastly, we used the SCORE2 Asia–Pacific algorithm to estimate the 10-year predicted CVD risk among individuals aged 40–69 years and without diabetes at baseline.^14^ We used the rescaling factors that corresponded to the low-risk region.^14^

### Discrimination and calibration

The discrimination of a model refers to its ability to distinguish between individuals at a higher risk and those at a lower risk. We evaluated the discrimination of the SG-FRS-2023, PCE, and SCORE2 Asia–Pacific model in the MEC1 and SEED cohorts using the concordance index (C-index). The C-index represents the probability of assigning a higher risk score to an individual with a shorter event-free survival duration compared to another randomly selected individual. A C-index <0.6 suggests poor discrimination, a C-index 0.60–0.75 suggests possibly helpful discrimination, and a C-index >0.75 suggests clearly useful discrimination.^20^

The calibration of a model refers to its ability to estimate the absolute risk of an event. We evaluated the calibration of the models using the calibration-in-the-large and calibration slope from a method detailed by Crowson et al.^21^ The calibration-in-the-large is an overall measure of the mean calibration with values close to zero indicating a satisfactory overall calibration. Calibration-in-the-large deviating from zero suggest a misestimation of risk (values greater than zero suggests an overall underestimation of risk, and values smaller than zero suggests an overall overestimation of risk).^21^ The calibration slope is a measure of the spread of calibration with values close to one indicating a satisfactory spread of calibration, and values deviating from one suggest a poor spread of calibration.^21^ When the calibration-in-the-large is close to zero and the calibration slope is close to one, it suggests that calibration is satisfactory across the entire sample.^22^

We evaluated the goodness-of-fit of the models using analysis of deviance.^21^ This involves adding a grouping variable to the model that predicts the outcome of interest using the predicted risk score and comparing the deviance against the base model, which only includes the predicted risk score as a predictor. The grouping variable was generated by categorising individuals into quintiles based on their predicted risk. We used quintiles such that at least 80% of the groups would have event counts of five or more.^23^ At a significance level of 5% and four degrees of freedom, deviance values > 9.5 suggest poor goodness-of-fit.

We also compared the observed and predicted risk across quintiles of predicted risk to visually assess the calibration and model fit using bar plots. To evaluate the clinical utility of the models, we used decision curve analysis to quantify the net benefit of the model across a range of threshold probabilities.^24^ All analyses were done using R (version 4.0.2) and R packages Survival (version 3.1–12) and Hmisc (version 4.4–0).

### Ethics approval

The MEC1 study was approved by the National University of Singapore Institutional Review Board (NUS-04-127 [7 December 2004], NUS-06-127 [1 December 2006]) and the SingHealth Centralised Institutional Review Board (2018/2970 [26 April 2009]). The SEED study was approved by the SingHealth Centralised Institutional Review Board (2018/2717 [3 August 2018], 2018/2921 [10 Oct 2018], 2012/487/A [24 July 2012], 2015/2279 [15 April 2015], 2018/2006 [14 June 2018], 2018/2594 [21 July 2018], 2018/2570 [2 July 2018]). All participants provided informed consent.

### Role of the funding source

The funders had no role in the study design, data collection, data analysis, interpretation, writing of the report, and the decision to submit the paper for publication.

## Results

Summary characteristics of MEC1 and the SEED cohort are shown in Supplementary Table S4. In the MEC1 cohort, the mean age of participants was 45.4 (±12.8) years at baseline; 43% of the participants were men, and the ethnic distribution was 47% Chinese, 26% Malay, and 27% Indian. In the SEED cohort, participants had a mean age of 58.1 (±10.2) years at baseline; 48% were male, and the ethnic distribution was 35% Chinese, 33% Malay, and 32% Indian. The mean follow-up duration was 11.9 (±2.6) years in MEC1 and 10.6 (±3.2) years in the SEED cohort.

### Discrimination

The C-index of the SG-FRS-2023 was 0.811 (95% CI 0.784–0.839) in MEC1 and 0.728 (95% CI 0.703–0.752) in the SEED cohort. The C-index of the PCE-W was 0.771 (95% CI 0.751–0.791) in MEC1 and 0.752 (95% CI 0.737–0.767) in the SEED cohort. In line with ACC/AHA recommendations,^7^ the original PCE-W tended to perform similarly or better than the PCE-AA across all ethnic groups in our study (Table 1). The C-index of the SCORE2 Asia–Pacific model was 0.786 (95% CI 0.758–0.815) in MEC1 and 0.745 (95% CI 0.718–0.771) in the SEED cohort.

**Table 1:** Concordance index (C-index) of the Singapore-modified Framingham Risk Score (SG-FRS-2023), Pooled Cohort Equations for Whites (PCE-W) and African Americans (PCE-AA), and SCORE2 Asia–Pacific model for MEC1 and SEED.

	SG-FRS-2023	PCE-W^a^	PCE-AA^a^	SCORE2 Asia–Pacific
**MEC1**				
Overall cohort	0.811 (0.784, 0.839)	0.771 (0.751, 0.791)	0.753 (0.734, 0.772)	0.786 (0.758, 0.815)
Chinese, male	0.822 (0.770, 0.874)	0.768 (0.726, 0.810)	0.773 (0.733, 0.813)	0.763 (0.703, 0.823)
Chinese, female	0.942 (0.906, 0.977)	0.860 (0.806, 0.913)	0.862 (0.811, 0.912)	0.864 (0.794, 0.934)
Malay, male	0.727 (0.578, 0.876)	0.750 (0.702, 0.798)	0.768 (0.723, 0.813)	0.779 (0.714, 0.843)
Malay, female	0.784 (0.718, 0.849)	0.799 (0.742, 0.855)	0.798 (0.742, 0.854)	0.792 (0.685, 0.899)
Indian, male	0.774 (0.719, 0.830)	0.696 (0.644, 0.748)	0.691 (0.641, 0.740)	0.692 (0.616, 0.769)
Indian, female	0.756 (0.668, 0.844)	0.715 (0.654, 0.775)	0.722 (0.663, 0.781)	0.724 (0.636, 0.812)
**SEED**				
Overall cohort	0.728 (0.703, 0.752)	0.752 (0.737, 0.767)	0.717 (0.701, 0.733)	0.745 (0.718, 0.771)
Chinese, male	0.771 (0.699, 0.842)	0.810 (0.774, 0.846)	0.793 (0.755, 0.830)	0.796 (0.740, 0.851)
Chinese, female	0.810 (0.727, 0.894)	0.815 (0.757, 0.874)	0.817 (0.759, 0.875)	0.772 (0.680, 0.863)
Malay, male	0.707 (0.657, 0.756)	0.706 (0.673, 0.738)	0.691 (0.658, 0.723)	0.718 (0.665, 0.771)
Malay, female	0.815 (0.745, 0.885)	0.787 (0.753, 0.820)	0.795 (0.762, 0.828)	0.767 (0.690, 0.843)
Indian, male	0.665 (0.612, 0.719)	0.688 (0.653, 0.723)	0.672 (0.636, 0.709)	0.649 (0.594, 0.704)
Indian, female	0.774 (0.688, 0.860)	0.778 (0.737, 0.819)	0.766 (0.725, 0.807)	0.720 (0.623, 0.816)

a The C-indices presented apply to both the original and recalibrated models, as the recalibration method used in this study does not affect the discrimination of the model.

Across both MEC1 and SEED, the C-indices were higher in women than men, and higher in Chinese than Malays and Indians in all three prediction models (Table 1). The sex- and ethnic-specific C-indices ranged from 0.649 (95% CI 0.594–0.704) for the SCORE2 Asia–Pacific model in Indian men to 0.942 (95% CI 0.906–0.977) for the SG-FRS-2023 in Chinese women.

### Calibration

**Fig. 2:**
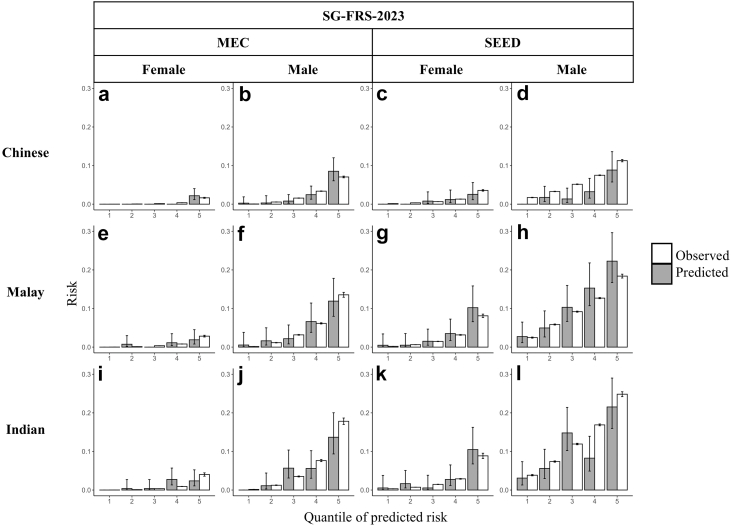
Comparison of the observed (grey) versus predicted (white) risks by the Singapore-modified Framingham Risk Score (SG-FRS-2023) in the Singapore Multi-Ethnic Cohort Phase 1 (MEC1) and Singapore Epidemiology of Eye Diseases study (SEED). Quintiles of observed (grey) and predicted (white) risk are presented by sex (columns) and ethnicity (rows). The first two columns (a, b, e, f, i, j) present observed and predicted risk for MEC1. The third and fourth columns (c, d, g, h, k, l) present observed and predicted risk in SEED. Error bars are 95% confidence intervals of the estimate.

The SG-FRS-2023 was generally satisfactorily calibrated in MEC1 and SEED, with some exceptions in the SEED cohort where an overestimation of risk was observed among Chinese men and Indian men (Fig. 2 and Supplementary Table S5).

**Fig. 3:**
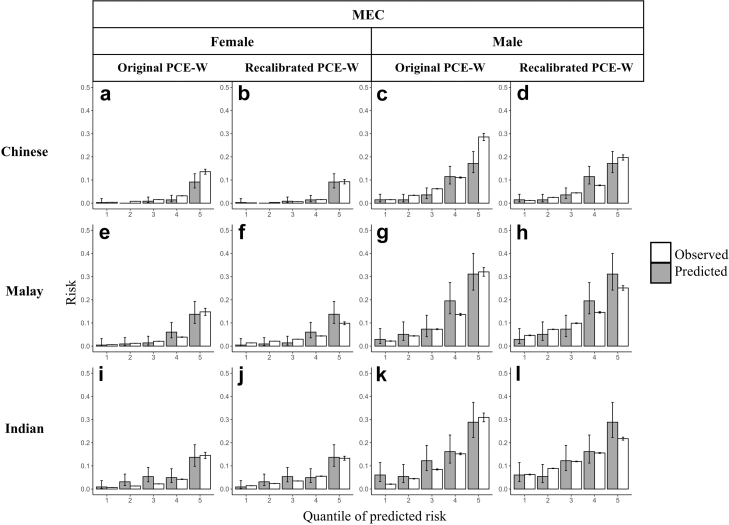
Comparison of the original Pooled Cohort Equations for Whites (PCE-W) versus recalibrated PCE-W in the Singapore Multi-Ethnic Cohort Phase 1 (MEC1). Quintiles of observed (grey) and predicted (white) risk are presented by sex (columns) and ethnicity (rows). The first and third columns (a, c, e, g, i, k) compare observed and predicted risk in the original PCE-W prediction model. The second and fourth columns (b, d, f, h, j, l) compare observed and predicted risk in the recalibrated PCE-W prediction model. Error bars are 95% confidence intervals of the estimate.

The original PCE-W displayed poor calibration in both MEC1 and SEED as it overestimated risk among Chinese and Malays and displayed poor calibration spread among Indians (Supplementary Table S6). After recalibration, both the mean and spread of calibration were improved across sex and ethnicity in MEC1 (Table 2 and Fig. 3). In the SEED cohort, the recalibrated PCE-W outperformed the original PCE-W, although some misestimations of risk were still observed among Chinese men, Chinese women, and Malay women (Supplementary Figure S1). Both the original and recalibrated PCE-W tended to perform similarly or better than the PCE-AA across all ethnic groups in our study (Supplementary Tables S7 and S8).

**Table 2:** Calibration of the recalibrated Pooled Cohort Equations for Whites (PCE-W) in the MEC1 (N = 6900) and SEED cohort (N = 8385).

	MEC1		SEED	
	Male	Female	Male	Female
**Chinese**				
N observed events^a^	99.4	42.3	125.4	50.4
N predicted events	100.8	43.0	163.2	75.3
Calibration-in-the-large^b^	−0.018 (−0.225, 0.175)	−0.046 (−0.368, 0.246)	−0.336 (−0.530, −0.154)	−0.479 (−0.786, −0.200)
Calibration slope^c^	0.944 (0.756, 1.135)	0.833 (0.605, 1.069)	1.450 (1.205, 1.702)	0.812 (0.556, 1.082)
Deviance^d^	8.2	3.0	30.7	13.5
**Malay**				
N observed events^a^	92.3	49.1	232.9	151.2
N predicted events	85.8	45.1	239.1	111.9
Calibration-in-the-large^b^	0.048 (−0.166, 0.248)	0.064 (−0.232, 0.335)	−0.102 (−0.239, 0.028)	0.260 (0.089, 0.422)
Calibration slope^c^	1.197 (0.989, 1.410)	1.183 (0.968, 1.397)	1.066 (0.921, 1.216)	1.194 (1.047, 1.348)
Deviance^d^	7.4	9.8	3.8	20.1
**Indian**				
N observed events^a^	103.8	63.3	207.6	110.3
N predicted events	97.4	59.1	201.8	115.4
Calibration-in-the-large^b^	0.019 (−0.183, 0.209)	0.053 (−0.206, 0.292)	−0.023 (−0.164, 0.113)	−0.086 (−0.283, 0.098)
Calibration slope^c^	1.185 (0.986, 1.386)	0.948 (0.741, 1.154)	1.254 (1.094, 1.416)	1.103 (0.938, 1.272)
Deviance^d^	5.1	3.2	12.6	4.9
**Overall cohort**				
N observed events^a^	449.3		879.6	
N predicted events	431.1		905.8	
Calibration-in-the-large^b^	0.020 (−0.075, 0.112)		−0.090 (−0.159, −0.022)	
Calibration slope^c^	1.045 (0.963, 1.128)		1.144 (1.075, 1.213)	
Deviance^d^	7.6		31.5	

a Adjusted using the Kaplan–Meier estimator to account for participants with follow-up duration <10 years.

b Calibration-in-the-large >0 indicate underestimation of risk, calibration-in-the-large <0 indicate overestimation of risk. Numbers in parentheses are the 95% confidence intervals of the estimate.

c Calibration slope ≠ 1 indicate poor spread of calibration. Numbers in parentheses are the 95% confidence intervals of the estimate.

d Deviance values > 9.5 indicates poor goodness-of-fit at a significance level of 5%.

The SCORE2 Asia–Pacific model overestimated risk among Chinese males and females in both MEC1 and SEED, with average predicted risks exceeding observed risks by more than twofold in the first three quintiles of predicted risk (Supplementary Table S9 and Supplementary Figure S2). Among Malay males and females, the SCORE2 Asia–Pacific model tended to overestimate risk among the lower risk groups while underestimating risk among the higher risk groups (Supplementary Figure S2), which resulted in a poor calibration spread and model fit.

### Clinical utility

All three models demonstrated higher net benefit than the “treat all” or “treat none” strategies across clinically relevant threshold probabilities (5–20%) in both cohorts (Supplementary Figure S3). The recalibrated PCE-W consistently showed the highest net benefit compared with SG-FRS-2023 and the SCORE2 Asia–Pacific model in both MEC1 and SEED cohorts.

## Discussion

This study had two main findings: (i) the PCE-W, SG-FRS-2023, and SCORE2 Asia–Pacific model demonstrated possibly helpful to clearly useful discrimination across sex and ethnicity in two independent cohorts. (ii) the SG-FRS-2023 and recalibrated PCE had better calibration compared to the SCORE2 Asia–Pacific model in our Singapore cohorts. Collectively, these results demonstrate that the SG-FRS-2023 and recalibrated PCE-W may be suitable for CVD risk assessment in Singapore.

While the discrimination of the PCE-W was satisfactory, the calibration of the original PCE-W in our Singapore cohorts was poor, with an overestimation of risk in Chinese and Malays and a poor calibration spread among Indians. In a previous study involving Chinese participants from three cohorts across various provinces in China, the PCE-W was found to overestimate the ASCVD risk in males while underestimating the ASCVD risk in females.^10^ Similarly, in another study among participants in the CHERRY study in China, the PCE-W overestimated 5-year ASCVD risk by 63% in males and underestimated risk by 34% in females.^9^ Our results are consistent with the overestimation of risk in Chinese males but not with the underestimation of risk in Chinese women observed in these Chinese cohorts. The PCE-W was previously reported to overestimate 10-year ASCVD risk in Chinese, Malay, and Indian ethnic groups in a Malaysian cohort.^13^ Our findings are in agreement with the overestimation of ASCVD risk in Chinese and Malays, but we did not observe overestimation of risk in Indians. These differences in the calibration of the PCE-W across populations are likely due to variations in risk factor distributions and CVD incidence rates, and underscores the importance of locally evaluating and recalibrating externally derived risk prediction models.

We recalibrated the PCE-W using data from MEC1 and externally validated the recalibrated models in the SEED cohort. The results show that the recalibrated PCE-W had an overall improved calibration compared to the original PCE-W in both cohorts. We further compared the calibration of the recalibrated PCE-W with the SG-FRS-2023 and SCORE2 Asia–Pacific model. The SG-FRS-2023 was generally satisfactorily calibrated across both MEC1 and the external SEED cohort. In contrast, the SCORE2 Asia–Pacific model consistently overestimated risk among Chinese and exhibited a poor model fit among Malays, overestimating risk in lower-risk individuals while underestimating risk in higher-risk individuals. Both the SG-FRS-2023 and PCE-W were recalibrated according to sex- and ethnic-specific survival data from MEC1. In comparison, the SCORE2 Asia–Pacific model uses sex-specific equations that are recalibrated using rescaling factors derived from different risk regions in the Asia–Pacific; in this case, the rescaling factors from the low-risk region were used. The use of region-specific rescaling factors instead of ethnic-specific incidence rates for recalibration may explain why the SG-FRS-2023 and recalibrated PCE-W tended to perform better than the SCORE2 Asia–Pacific model in our multi-ethnic Singapore cohorts, considering that substantial ethnic differences in CVD risk between Asian ethnic groups in Singapore have been previously documented.^16^^,^^17^ It is important to emphasise that ethnicity is a sociocultural construct rather than a biological trait. The inclusion of ethnicity in CVD prediction models should therefore be interpreted as a proxy for unmeasured structural, socioeconomic, and environmental factors that influence cardiovascular risk. We did not evaluate rescaling factors derived from the moderate- and high-risk regions in the SCORE2 Asia–Pacific model, as the rescaling factors for the low-risk region already led to an overestimation of risk among Chinese participants in both MEC1 and the SEED cohort.

The three evaluated models were each developed to predict a different set of outcomes in the general population. The SG-FRS-2023 predicts the 10-year risk of CHD; the PCE-W predicts the 10-year risk of ASCVD that includes CHD and stroke; and the SCORE2 Asia–Pacific model predicts the 10-year risk of CVD that includes CHD, stroke, hypertensive diseases, arrhythmias, heart failure, and atherosclerosis. Unlike the SG-FRS-2023 and SCORE2 Asia–Pacific model, which do not include individuals with diabetes, the PCE-W included diabetes as a predictor and was designed for risk assessment among individuals with or without diabetes. Our study, together with others, has shown that all three established models generally demonstrate possibly helpful to clearly useful discrimination (C-statistics >0.60) in Asian populations with diverse ethnic compositions.^8^, ^9^, ^10^, ^11^^,^^14^^,^^25^, ^26^, ^27^, ^28^ As demonstrated in this study and previously by others, the calibration of prediction models can be substantially improved after local recalibration.^29^^,^^30^ Beyond discrimination and calibration, the decision curve analysis further demonstrated the clinical utility of the three models. Across clinically relevant threshold probabilities (5–20%), all three models showed higher net benefit than the “treat all” or “treat none” strategies, suggesting their usefulness for guiding primary prevention decisions. Hence, the choice of a specific model may ultimately depend on the outcome of interest and the target population, assuming a local recalibration is performed when necessary. Here, the recalibrated PCE-W consistently exhibited the highest net benefit in both Singapore cohorts. This is likely attributable to its inclusion of individuals with diabetes, and its broader outcome definition. Given that ASCVD is the leading cause of CVD mortality in Singapore,^31^ the recalibrated PCE-W may be the preferred model for CVD risk assessment in Singapore.

Our study has several strengths. We included data from ethnic Indians (South Asian) and Malays (Southeast Asian), which are populations that have been underrepresented in CVD prediction research. Furthermore, oversampling of ethnic minorities (Malay and Indians) was done in both cohorts to ensure adequate representation across the three major ethnic groups in Singapore. Next, both of our cohorts had an average follow-up over 10 years, which allowed us to assess the performance of the models with minimal extrapolation. In addition, the ascertainment of CVD incidence in our study was virtually complete, as it was obtained from national registries.

We acknowledge the potential limitations of our study. First, to conduct analyses in each sex and ethnic group separately, the number of events in each subgroup was reduced, which resulted in the use of quintiles for the test of model fit instead of the usual deciles. As a result, the model fit estimates may be conservative. In addition, the smaller number of events in each subgroup may lead to larger variability and lower precision. Second, compared to the MEC1 cohort, the SEED cohort participants were older at baseline and were more likely to be diagnosed with diabetes and hypertension. This heterogeneity across the MEC1 (development) and SEED (validation) cohorts may partly explain the differences in the calibration of the SG-FRS-2023 and recalibrated PCE-W across both cohorts. Lastly, we did not assess the generalisability of our findings to populations outside Singapore. Therefore, the recalibrated PCE-W in this study may not be fully generalisable to other populations, and we strongly recommend that any externally derived prediction model be locally evaluated before adoption.

In conclusion, the recalibrated PCE-W and SG-FRS-2023 demonstrated possibly helpful to clearly useful discrimination and generally satisfactory calibration in two multi-ethnic cohorts in Singapore. These models can support clinicians in stratifying individuals into different cardiovascular risk groups, which may in turn guide the intensity of preventive strategies in accordance with existing clinical guidelines. Between the two, the recalibrated PCE-W may be preferred over the SG-FRS-2023 for CVD risk assessment in Singapore, as it predicts ASCVD events and is applicable to individuals with diabetes. The SCORE2 Asia–Pacific model also showed possibly helpful to clearly useful discrimination, but it requires recalibration before being applied in the local context. Future studies are required to evaluate the use of the SCORE2 Asia–Pacific model in other multi-ethnic populations in the Asia–Pacific region.

## Contributors

Conceptualisation: XS, CYC, RVD, SH, JCC, KKY, EST. Funding acquisition: XS, CYC, RVD, JCC, KKY. Methodology: CGYL, SM. Formal analysis: CGYL, CCYC, YHMW, JY. Supervision: XS, CS, SH, RVD, JT. Validation: CCYC, CS, CYC. Writing—original draft: CGYL, XS. Writing—review & editing: all authors.

XS and CGYL has directly accessed and verified the underlying data reported in the manuscript and were responsible for the decision to submit the manuscript.

## Data sharing statement

The linked datasets (exposure and outcomes) used in this study are analysed in a secure environment and are not available for external request.

## Declaration of interests

CYC has received consulting fees from Mediwhale and is a co-founder of Eye AI. JT has received grants, contracts, or consulting fees from AstraZeneca, Roche Diagnostics, Us2. ai, and the Asian Development Bank, and owns a patent US-10702247-B2 unrelated to the present work.
