# Supplementary material for: Cardiovascular disease risk prediction in multi-ethnic Asian populations: evidence from two population-based cohorts in Singapore
Source: Lancet Reg Health West Pac. 2026 Jan 2;66:101794. doi: 10.1016/j.lanwpc.2025.101794 (PMC12805090; doi:10.1016/j.lanwpc.2025.101794)
Supplement: Supplementary Materials [file mmc1.docx]

**Supplementary Materials**

Table of Contents

[Supplementary Note 2](#_Toc214909346)

[**Supplementary Note 1. Recalibration of the Pooled Cohort Equations using a modified SCORE2 recalibration method** 2](#_Toc214909347)

[Supplementary Tables 6](#_Toc214909348)

[**Supplementary Table 1. Summary of the three CVD prediction models evaluated in this study.** 6](#_Toc214909349)

[**Supplementary Table 2. Rescaling factors for the recalibration of the Pooled Cohort Equations for Whites (PCE-W) by sex and ethnic groups.** 7](#_Toc214909350)

[**Supplementary Table 3. Equation parameters of the Pooled Cohort Equations for Whites (PCE-W) according to sex.** 8](#_Toc214909351)

[**Supplementary Table 4. Baseline characteristics of participants in MEC1 and SEED before further exclusion for age and diabetes status that are specific to each evaluated prediction model.** 9](#_Toc214909352)

[**Supplementary Table 5. Calibration of the Singapore-modified Framingham Risk Score (SG-FRS-2023) in the MEC1 (N = 8,722) and SEED cohort (N = 6,132).** 10](#_Toc214909353)

[**Supplementary Table 6. Calibration of the original Pooled Cohort Equations for Whites (PCE-W) in the MEC1 (N = 6,900) and SEED cohort (N = 8,385).** 11](#_Toc214909354)

[**Supplementary Table 7. Calibration of the original Pooled Cohort Equations for African Americans (PCE-AA) in the MEC1 (N = 6,900) and SEED cohort (N = 8,385).** 12](#_Toc214909355)

[**Supplementary Table 8. Calibration of the recalibrated Pooled Cohort Equations for African Americans (PCE-AA) in the MEC1 (N = 6,900) and SEED cohort (N = 8,385).** 13](#_Toc214909356)

[**Supplementary Table 9. Calibration of the SCORE2 Asia-Pacific model in the MEC1 (N = 5,447) and SEED cohort (N = 5,359).** 14](#_Toc214909357)

[Supplementary Figures 15](#_Toc214909358)

[**Supplementary Figure 1. Comparison of the original Pooled Cohort Equations for Whites (PCE-W) versus recalibrated PCE-W in the Singapore Epidemiology of Eye Diseases study (SEED).** 15](#_Toc214909359)

[**Supplementary Figure 2. Comparison of the observed (grey) versus predicted (white) risks by the SCORE2 Asia-Pacific model (SCORE2-AP) in the Singapore Multi-Ethnic Cohort Phase 1 (MEC1) and Singapore Epidemiology of Eye Diseases study (SEED).** 16](#_Toc214909360)

[**Supplementary Figure 3. Decision curves showing net benefit across a range of threshold probabilities for CVD prediction using the Pooled Cohort Equations for Whites (PCE-W) and African Americans (PCE-AA), Singapore-modified Framingham Risk Score (SG-FRS-2023), and SCORE2 Asia-Pacific model (SCORE2-AP) in the Singapore Multi-Ethnic Cohort Phase 1 (MEC1) and Singapore Epidemiology of Eye Diseases study (SEED).** 17](#_Toc214909361)

# **Supplementary Note**

## **Supplementary Note 1. Recalibration of the Pooled Cohort Equations using a modified SCORE2 recalibration method**

The original SCORE2 recalibration method is described in detail elsewhere (1,2). Briefly, it involves the estimation of the sex, ethnic, and age-specific average expected risk and the average predicted risk in the target population. The average expected risk is then related to the average predicted risk to obtain a set of rescaling factors. Finally, the rescaling factors are applied to an individual estimated risk to obtain the recalibrated 10-year predicted risk.

In the original SCORE2 method, the sex and age-specific 10-year average expected risk was estimated through the following steps.

1. The 1-year fatal ASCVD rate was obtained from national statistics.
2. The 1-year fatal ASCVD rate was extrapolated to 10-year fatal ASCVD risk using the life table method, with adjustment for competing risk of non-CVD death.
3. As the outcome of interest is any first occurring ASCVD event that includes both fatal and non-fatal events, there is a need to translate the 10-year ASCVD mortality risk into 10-year ASCVD incidence that includes non-fatal acute myocardial infarction and stroke. This was done using multiplication factors estimated in nationally representative cohorts. The multiplication factor was calculated as the 10-year risk of total ASCVD divided by the 10-year risk of fatal ASCVD in the cohort. The denominator includes the entire cohort to reflect the fact that the population statistics were calculated among the whole population. The numerator includes participants without pre-existing CVDs to reflect the target population for primary prevention.

*Multiplication factor* = $\frac{{10-year risk of total ASCVD}_{without prior CVD}}{{10-year risk of fatal ASCVD}_{entire population}}$

1. The 10-year expected ASCVD risk was calculated as the 10-year fatal ASCVD risk from step 2 multiplied by the multiplication factor from step 3.

We apply the SCORE2 recalibration method to the Pooled Cohort Equations. There are 4 steps to this recalibration method. Steps 2 to 4 are unchanged from the original SCORE2 recalibration method. Step 1 is modified from the SCORE2 recalibration method.

- Step 1 involves the estimation of the sex, ethnic, and age specific average 10-year expected risk in the target population.
- Step 2 involves the estimation of the sex, ethnic, and age specific average 10-year predicted risk in the target population.
- Step 3 involves the derivation of sex- and ethnic-specific rescaling factors by regressing the average 10-year expected risk against the average 10-year predicted risk separately for each sex and ethnic group.
- Step 4 involves the application of the rescaling factors to an individual 10-year predicted risk to obtain the recalibrated 10-year predicted risk for an individual. Each step is described in detail below.

As we need to perform recalibration separately for the three major ethnic groups in Singapore (Chinese, Malay, Indian) and further stratify by sex and age subgroups, the sample size in each subgroup in our cohort was limited. This led to strata where there were less than 5 fatal ASCVD event in certain subgroups (particularly for younger age groups and females) despite using 10-year age categories instead of 5-year age categories to increase the sample size in each subgroup. The small number of fatal ASCVD events in each subgroup limited our ability to reliably estimate the multiplication factor as even small variations in the number of fatal ASCVD events (denominator) would have a considerably large impact on the multiplication factor. In instances when the number of fatal ASCVD events in a specific subgroup was zero, the multiplication factor could not be estimated.

To overcome this limitation, we used the Kaplan-Meier estimator to estimate the sex, ethnic, and age-specific 10-year total ASCVD risk (which include both fatal and non-fatal events) in a population-based cohort, the Singapore Multi-Ethnic Cohort Phase 1 (MEC1). This allowed us to estimate the 10-year expected ASCVD risk without the need for the estimation of multiplication factors.

Step 1: Obtain the average 10-year expected risk in the target population.

The average 10-year expected ASCVD risk was estimated from a population-based cohort, the Singapore Multi-Ethnic Cohort Phase 1 (MEC1), using the Kaplan-Meier estimator. This was done within each sex, ethnic, and 10-year age group.

Step 2: Obtain the average 10-year predicted risk in the target population.

The average predicted 10-year ASCVD risk was estimated using the means of the risk factors in MEC1 and the parameters (coefficients and baseline survival) from the PCE. This was done within each sex, ethnic, and 10-year age group.

$$Average predicted 10year ASCVD risk= 1- {S_{10,PCE}}^{exp(\sum\beta_{PCE}\left( \bar{x}_{MEC}-\bar{x}_{PCE} \right))}$$

where:

$S_{10,PCE}$ is the average 10-year survival from the PCE

$\beta_{PCE}$ are the coefficients from the PCE

$\bar{x}_{PCE}$ are the means of the risk factors from the PCE

$\bar{x}_{MEC1}$ are the means of the risk factors in the target population estimated from the Singapore Multi-Ethnic Cohort Phase 1

Step 3: Derive rescaling factors for each sex and ethnic group.

Within each sex and ethnic group, the age-specific expected 10-year ASCVD risk was regressed on the age-specific predicted 10-year ASCVD risk to obtain **scale1** (intercept) and **scale2** (beta). The scaling factors are reported **in Supplementary Table 2.**

$$ln(-\ln\left( 1-average expected risk \right)) \sim\boldsymbol{scale}\boldsymbol{1}+\boldsymbol{scale}\boldsymbol{2}\times ln(-ln(1-average predicted risk)$$

Step 4: Calculated the recalibrated 10-year ASCVD risk for an individual

First, the (uncalibrated) 10-year ASCVD risk was calculated using the PCE using the formula

$$Uncalibrated 10Y ASCVD risk= 1- {S_{10,PCE}}^{exp(\sum\beta_{PCE}\left( x_{individual}-\bar{x}_{PCE} \right))}$$

where:

$S_{10,PCE}$ is the average 10-year survival from the PCE

$\beta_{PCE}$ are the coefficients from the PCE

$\bar{x}_{PCE}$ are the means of the risk factors from the PCE

$x_{individual}$ are values of the risk factors for an individual

Subsequently, the recalibrated 10-year ASCVD risk for an individual is calculated using the rescaling factors from Step 3:

$$Recalibrated 10Y ASCVD risk = 1-exp(-\exp\left( \boldsymbol{scale}\boldsymbol{1}+\boldsymbol{scale}\boldsymbol{2}\times\ln\left( -\ln\left( 1-10Y ASCVD risk \right) \right) \right))$$

**Worked example**

A worked example is shown below. A Chinese woman, aged 55 years, has a total cholesterol level of 4 mmol/L (154·7 mg/dL), HDL-cholesterol level of 1 mmol/L (38·67 mg/dL), systolic blood pressure of 140 mmHg and is on hypertension medication, is a current smoker, and does not have diabetes. Using the original PCE-W coefficients for women (3) (**Supplementary Table 3)**, the uncalibrated 10-year ASCVD risk was calculated to be 8%. Finally, using the rescaling factors from **Supplementary Table 2**, the recalibrated 10-year predicted ASCVD risk for this individual was estimated to be 5%.

|  | **Coefficient** | **Individual example value** | **Coefficient × Value** |
| --- | --- | --- | --- |
| **Ln Age (y)** | -29·799 | 4·01 | -119·41 |
| **Ln Age, Squared** | 4·884 | 16·06 | 78·43 |
| **Ln Total Cholesterol (mg/dL)** | 13·54 | 5·04 | 68·26 |
| **Ln Age × Ln Total Cholesterol** | -3·114 | 20·20 | -62·91 |
| **Ln HDL-C (mg/dL)** | -13·578 | 3·66 | -49·63 |
| **Ln Age × Ln HDL-C** | 3·149 | 14·65 | 46·12 |
| **Ln Treated Systolic BP (mm Hg)** | 2·019 | 4·94 | 9·98 |
| **Ln Untreated Systolic BP (mm Hg)** | 1·957 | NA | NA |
| **Current Smoker (1=Yes, 0=No)** | 7·574 | 1 | 7·57 |
| **Ln Age × Current Smoker** | -1·665 | 4·01 | -6·67 |
| **Diabetes (1=Yes, 0=No)** | 0·661 | 0 | 0 |
| **Individual sum** |  |  | -28·26 |
| **Mean (Coefficient × Value)** |  |  | -29·18 |
| **Average 10-year survival** | 0·9665 |  |  |
| **10-year ASCVD risk** | $= 1- {S_{10,PCE}}^{\exp\left( \sum\beta_{PCE}\left( x_{individual}-\bar{x}_{PCE} \right) \right)}$  $= 1- {0\cdot9665}^{\exp\left( -28\cdot26- -29\cdot18 \right)}$  $= 0\cdot08$ | | |
| **Recalibrated 10-year ASCVD risk** | $= 1-exp(-\exp\left( -0\cdot14022+1\cdot175498\times\ln\left( -\ln\left( 1-0\cdot08 \right) \right) \right))$  $= 0\cdot05$ | | |

**References**

1. SCORE2 working group and ESC Cardiovascular risk collaboration. SCORE2 risk prediction algorithms: new models to estimate 10-year risk of cardiovascular disease in Europe. Eur Heart J. 2021 Jul 1;42(25):2439–54.

2. Hageman SHJ, Huang Z, Lee H, Kaptoge S, Dorresteijn JAN, Pennells L, et al. Risk prediction of cardiovascular disease in the Asia-Pacific region: the SCORE2 Asia-Pacific model. Eur Heart J. 2025 Feb;46(8):702–15.

3. Goff DCJ, Lloyd-Jones DM, Bennett G, Coady S, D’Agostino RB, Gibbons R, et al. 2013 ACC/AHA guideline on the assessment of cardiovascular risk: a report of the American College of Cardiology/American Heart Association Task Force on Practice Guidelines. Circulation. 2014 Jun;129(25 Suppl 2):S49-73.

# **Supplementary Tables**

## **Supplementary Table 1. Summary of the three CVD prediction models evaluated in this study.**

|  | **SG-FRS-2023** | **PCE-W and Recalibrated PCE-W** | **SCORE2 Asia-Pacific** |
| --- | --- | --- | --- |
| **Original model** | Framingham-based ATP III model developed from the Framingham Heart Study and Framingham Offspring Study. | PCE developed in racially and geographically diverse US populations. | SCORE2 model developed using data from using data from 13 European countries. |
| **Intended to predict** | CHD | ASCVD (CHD and stroke) | CVD (including CHD, stroke, hypertensive diseases, arrhythmias, heart failure, and atherosclerosis) |
| **Predictors** | - age - total cholesterol - HDL cholesterol - systolic blood pressure - hypertension treatment - smoking history | - age - total cholesterol - HDL cholesterol - systolic blood pressure - hypertension treatment - smoking history - diabetes status | - age - total cholesterol - HDL cholesterol - systolic blood pressure - hypertension treatment - smoking history |
| **Interaction terms** | - age * age (male only) - age * total cholesterol - age * smoking history | - age * age (female only) - age * total cholesterol - age * HDL cholesterol - age * smoking history | - age * total cholesterol - age * HDL cholesterol - age * systolic blood pressure - age * smoking history |
| **Age range** | 20-79 | 40-79 | 40-69 |
| **Include individuals with diabetes** | No | Yes | No |
| **Recalibrated using data from** | MEC1 and linkage with national registries up till Dec 2017. | MEC1 and linkage with national registries up till Dec 2019. | Country-specific WHO GHE mortality data, country-specific risk factors from NCD RisC, and multipliers derived from 5 countries in the Asia-Pacific region, including South Korea, Singapore, Thailand, Brunei, and China. |

Abbreviations: ASCVD, atherosclerotic cardiovascular diseases; ATP, Adult Treatment Panel; CHD, coronary heart diseases; CVD, cardiovascular diseases; MEC1, Singapore Multi-Ethnic Cohort Phase 1; NCD RisC Non-Communicable Disease Risk Factor Collaboration; PCE-W, Pooled Cohort Equations for Whites; SCORE2, Systematic COronary Risk Evaluation 2; SG-FRS-2023, Singapore-modified Framingham Risk Score; WHO GHE, World Health Organization’s Global Health Estimates.

## **Supplementary Table 2. Rescaling factors for the recalibration of the Pooled Cohort Equations for Whites (PCE-W) by sex and ethnic groups.**

| **Ethnicity** | **Sex** | **Scale 1** | **Scale 2** |
| --- | --- | --- | --- |
| Chinese | Male | -0·50715 | 0·941062 |
| Malay | Male | -0·63675 | 0·628843 |
| Indian | Male | -0·93678 | 0·463546 |
| Chinese | Female | -0·14022 | 1·175498 |
| Malay | Female | -1·08174 | 0·625385 |
| Indian | Female | -0·55963 | 0·731909 |

## **Supplementary Table 3. Equation parameters of the Pooled Cohort Equations for Whites (PCE-W) according to sex.**

|  | **Female** | **Male** |
| --- | --- | --- |
| **Coefficients for risk factors** |  |  |
| Ln Age (y) | -29·799 | 12·344 |
| Ln Age, Squared | 4·884 | NA |
| Ln Total Cholesterol (mg/dL) | 13·54 | 11·853 |
| Ln Age × Ln Total Cholesterol | -3·114 | -2·664 |
| Ln HDL-C (mg/dL) | -13·578 | -7·990 |
| Ln Age × Ln HDL-C | 3·149 | 1·769 |
| Ln Treated Systolic BP (mm Hg) | 2·019 | 1·797 |
| Ln Untreated Systolic BP (mm Hg) | 1·957 | 1·764 |
| Current Smoker (1=Yes, 0=No) | 7·574 | 7·837 |
| Ln Age × Current Smoker | -1·665 | -1·795 |
| Diabetes (1=Yes, 0=No) | 0·661 | 0·658 |
| **Sum of coefficient × mean risk factor** | -29·18 | 61·18 |
| **Average 10-year survival** | 0·9665 | 0·9144 |

Values are reproduced from Goff et al. 2013 ACC/AHA guideline on the assessment of cardiovascular risk: a report of the American College of Cardiology/American Heart Association Task Force on Practice Guidelines. Circulation. 2014 Jun;129(25 Suppl 2):S49-73.

## **Supplementary Table 4. Baseline characteristics of participants in MEC1 and SEED before further exclusion for age and diabetes status that are specific to each evaluated prediction model.**

| **Characteristics** | **MEC1** | | | | **SEED** | | | |
| --- | --- | --- | --- | --- | --- | --- | --- | --- |
|  | **Chinese** | **Malay** | **Indian** | **All** | **Chinese** | **Malay** | **Indian** | **All** |
| **N** | 4619 | 2677 | 2840 | 10136 | 2964 | 2832 | 2769 | 8565 |
| **Male** | 2065 (44·7) | 1091 (40·8) | 1179 (41·5) | 4335 (42·8) | 1431 (48·3) | 1323 (46·7) | 1317 (47·6) | 4071 (47·5) |
| **Age, years** | 46·4 ± 12·9 | 44·3 ± 12·8 | 44·8 ± 12·7 | 45·4 ± 12·8 | 58·9 ± 9·6 | 58·7 ± 11·0 | 56·6 ± 9·7 | 58·1 ± 10·2 |
| **History of diabetes** | 369 (8·0) | 406 (15·2) | 624 (22·0) | 1399 (13·8) | 478 (16·1) | 847 (29·9) | 988 (35·7) | 2313 (27·0) |
| **Smoker** | 495 (10·7) | 611 (22·8) | 481 (16·9) | 1587 (15·7) | 372 (12·6) | 581 (20·5) | 399 (14·4) | 1352 (15·8) |
| **Hypertension medication** | 549 (11·9) | 192 (7·2) | 244 (8·6) | 985 (9·7) | 955 (32·2) | 747 (26·4) | 845 (30·5) | 2547 (29·7) |
| **SBP, mmHg** | 127 ± 20·3 | 128 ± 20·8 | 124 ± 21·2 | 126 ± 20·8 | 136 ± 19·3 | 147 ± 24·2 | 135 ± 20·1 | 140 ± 22·0 |
| **TC, mmol/L** | 5·21 ± 0·93 | 5·48 ± 1·06 | 5·15 ± 1·00 | 5·26 ± 0·99 | 5·51 ± 1·04 | 5·67 ± 1·14 | 5·31 ± 1·08 | 5·50 ± 1·10 |
| **HDL, mmol/L** | 1·43 ± 0·37 | 1·23 ± 0·32 | 1·12 ± 0·31 | 1·29 ± 0·36 | 1·33 ± 0·40 | 1·36 ± 0·33 | 1·08 ± 0·32 | 1·26 ± 0·37 |
| **Follow-up duration, years** | 12·5 ± 2·6 | 11·5 ± 2·4 | 11·2 ± 2·5 | 11·9 ± 2·6 | 9·13 ± 1·8 | 12·2 ± 3·9 | 10·6 ± 2·7 | 10·6 ± 3·2 |

Numbers are counts (percentage) or mean ± SD

Abbreviations: MEC1, Singapore Multi-Ethnic Cohort Phase 1; SEED, Singapore Epidemiology of Eye Diseases study

## **Supplementary Table 5. Calibration of the Singapore-modified Framingham Risk Score (SG-FRS-2023) in the MEC1 (N = 8,722) and SEED cohort (N = 6,132).**

|  | **MEC1** | | **SEED** | |
| --- | --- | --- | --- | --- |
|  | **Male** | **Female** | **Male** | **Female** |
|  | **Chinese** | | | |
| **N observed events^a^** | 46·2 | 10·2 | 35·1 | 11·5 |
| **N predicted events** | 47·2 | 10·5 | 67·0 | 15·2 |
| **Calibration-in-the-large^b^** | -0·028 (-0·339, 0·253) | -0·006 (-0·697, 0·556) | -0·671 (-1·033, -0·348) | -0·296 (-0·951, 0·242) |
| **Calibration slope^c^** | 1·179 (0·797, 1·611) | 1·455 (0·917, 2·030) | 1·685 (1·037, 2·394) | 1·094 (0·486, 1·802) |
| **Deviance^d^** | 6·1 | 6·3 | 28·1 | 3·5 |
|  | **Malay** | | | |
| **N observed events^a^** | 42·5 | 10·1 | 102·9 | 33·5 |
| **N predicted events** | 44·9 | 11·1 | 89·9 | 28·1 |
| **Calibration-in-the-large^b^** | -0·072 (-0·394, 0·219) | -0·078 (-0·769, 0·484) | 0·134 (-0·075, 0·328) | 0·166 (-0·215, 0·504) |
| **Calibration slope^c^** | 0·939 (0·602, 1·318) | 0·576 (0·142, 1·087) | 1·145 (0·791, 1·525) | 1·130 (0·745, 1·563) |
| **Deviance^d^** | 2·7 | 6·9 | 2·9 | 1·5 |
|  | **Indian** | | | |
| **N observed events^a^** | 47·1 | 15·4 | 87·8 | 29·6 |
| **N predicted events** | 55·0 | 14·2 | 106·9 | 26·6 |
| **Calibration-in-the-large^b^** | -0·184 (-0·491, 0·095) | 0·092 (-0·460, 0·559) | -0·214 (-0·434, -0·009) | 0·098 (-0·297, 0·447) |
| **Calibration slope^c^** | 0·886 (0·588, 1·222) | 0·605 (0·274, 0·975) | 0·914 (0·571, 1·279) | 0·865 (0·531, 1·218) |
| **Deviance^d^** | 6·3 | 8·9 | 12·7 | 3·6 |
|  | **Overall cohort** | | | |
| **N observed events^a^** | 170·7 | | 298·1 | |
| **N predicted events** | 182·5 | | 333·6 | |
| **Calibration-in-the-large^b^** | -0·076 (-0·232, 0·073) | | -0·122 (-0·241, -0·007) | |
| **Calibration slope^c^** | 0·923 (0·766, 1·087) | | 1·110 (0·947, 1·274) | |
| **Deviance^d^** | 3·2 | | 12·5 | |
| ^a^Adjusted using the Kaplan-Meier estimator to account for participants with follow-up duration < 10 years  ^b^Calibration-in-the-large >0 indicate underestimation of risk, calibration-in-the-large <0 indicate overestimation of risk. Numbers in parentheses are the 95% confidence intervals of the estimate.  ^c^Calibration slope ≠ 1 indicate poor spread of calibration. Numbers in parentheses are the 95% confidence intervals of the estimate.  ^d^Deviance values > 9·5 indicates poor goodness-of-fit at a significance level of 5%. | | | | |

## **Supplementary Table 6. Calibration of the original Pooled Cohort Equations for Whites (PCE-W) in the MEC1 (N = 6,900) and SEED cohort (N = 8,385).**

|  | **MEC1** | | **SEED** | |
| --- | --- | --- | --- | --- |
|  | **Male** | **Female** | **Male** | **Female** |
|  | **Chinese** | | | |
| **N observed events^a^** | 99·4 | 42·3 | 125·4 | 50·4 |
| **N predicted events** | 144·0 | 70·6 | 236·1 | 113·5 |
| **Calibration-in-the-large^b^** | -0·422 (-0·629, -0·229) | -0·568 (-0·891, -0·277) | -0·758 (-0·952, -0·576) | -0·930 (-1·237, -0·651) |
| **Calibration slope^c^** | 0·889 (0·700, 1·080) | 1·005 (0·777, 1·240) | 1·395 (1·150, 1·647) | 0·984 (0·728, 1·254) |
| **Deviance^d^** | 28·7 | 20·2 | 96·0 | 54·4 |
|  | **Malay** | | | |
| **N observed events^a^** | 92·3 | 49·1 | 232·9 | 151·2 |
| **N predicted events** | 83·3 | 49·2 | 281·7 | 166·5 |
| **Calibration-in-the-large^b^** | 0·094 (-0·120, 0·294) | -0·048 (-0·345, 0·222) | -0·280 (-0·417, -0·149) | -0·167 (-0·338, -0·005) |
| **Calibration slope^c^** | 0·853 (0·643, 1·067) | 0·821 (0·604, 1·036) | 0·728 (0·582, 0·879) | 0·841 (0·693, 0·995) |
| **Deviance^d^** | 4·0 | 3·7 | 29·1 | 7·8 |
|  | **Indian** | | | |
| **N observed events^a^** | 103·8 | 63·3 | 207·6 | 110·3 |
| **N predicted events** | 92·5 | 51·8 | 226·1 | 116·8 |
| **Calibration-in-the-large^b^** | 0·098 (-0·104, 0·288) | 0·187 (-0·072, 0·426) | -0·121 (-0·263, 0·014) | -0·111 (-0·308, 0·073) |
| **Calibration slope^c^** | 0·683 (0·483, 0·886) | 0·688 (0·480, 0·895) | 0·765 (0·603, 0·929) | 0·845 (0·680, 1·014) |
| **Deviance^d^** | 10·5 | 12·1 | 13·3 | 3·9 |
|  | **Overall cohort** | | | |
| **N observed events^a^** | 449·3 | | 879·6 | |
| **N predicted events** | 491·7 | | 1141·7 | |
| **Calibration-in-the-large^b^** | -0·121 (-0·216, -0·029) | | -0·336 (-0·406, -0·268) | |
| **Calibration slope^c^** | 0·827 (0·744, 0·911) | | 0·896 (0·826, 0·966) | |
| **Deviance^d^** | 19·9 | | 111·4 | |
| ^a^Adjusted using the Kaplan-Meier estimator to account for participants with follow-up duration < 10 years  ^b^Calibration-in-the-large >0 indicate underestimation of risk, calibration-in-the-large <0 indicate overestimation of risk. Numbers in parentheses are the 95% confidence intervals of the estimate.  ^c^Calibration slope ≠ 1 indicate poor spread of calibration. Numbers in parentheses are the 95% confidence intervals of the estimate.  ^d^Deviance values > 9·5 indicates poor goodness-of-fit at a significance level of 5%. | | | | |

## **Supplementary Table 7. Calibration of the original Pooled Cohort Equations for African Americans (PCE-AA) in the MEC1 (N = 6,900) and SEED cohort (N = 8,385).**

|  | **MEC1** | | **SEED** | |
| --- | --- | --- | --- | --- |
|  | **Male** | **Female** | **Male** | **Female** |
|  | **Chinese** | | | |
| **N observed events^a^** | 99·4 | 42·3 | 125·4 | 50·4 |
| **N predicted events** | 153·6 | 104·8 | 222·8 | 147·3 |
| **Calibration-in-the-large^b^** | -0·476 (-0·683, -0·282) | -0·983 (-1·306, -0·692) | -0·684 (-0·877, -0·502) | -1·186 (-1·493, -0·907) |
| **Calibration slope^c^** | 1·112 (0·885, 1·338) | 1·101 (0·829, 1·395) | 1·418 (1·167, 1·672) | 1·294 (0·936, 1·686) |
| **Deviance^d^** | 44·0 | 62·2 | 84·1 | 101·5 |
|  | **Malay** | | | |
| **N observed events^a^** | 92·3 | 49·1 | 232·9 | 151·2 |
| **N predicted events** | 79·4 | 80·4 | 251·0 | 238·3 |
| **Calibration-in-the-large^b^** | 0·160 (-0·054, 0·360) | -0·575 (-0·872, -0·305) | -0·137 (-0·274, -0·006) | -0·569 (-0·740, -0·407) |
| **Calibration slope^c^** | 1·103 (0·857, 1·349) | 0·835 (0·601, 1·082) | 0·804 (0·636, 0·974) | 1·030 (0·845, 1·227) |
| **Deviance^d^** | 8·7 | 23·2 | 14·0 | 57·7 |
|  | **Indian** | | | |
| **N observed events^a^** | 103·8 | 63·3 | 207·6 | 110·3 |
| **N predicted events** | 88·7 | 81·5 | 213·5 | 164·6 |
| **Calibration-in-the-large^b^** | 0·160 (-0·043, 0·349) | -0·311 (-0·571, -0·072) | -0·055 (-0·196, 0·081) | -0·480 (-0·677, -0·295) |
| **Calibration slope^c^** | 0·829 (0·582, 1·074) | 0·660 (0·452, 0·876) | 0·787 (0·607, 0·967) | 0·938 (0·734, 1·152) |
| **Deviance^d^** | 6·8 | 17·1 | 6·1 | 32·6 |
|  | **Overall cohort** | | | |
| **N observed events^a^** | 447·5 | | 872·1 | |
| **N predicted events** | 588·7 | | 1237·4 | |
| **Calibration-in-the-large^b^** | -0·310 (-0·405, -0·218) | | -0·419 (-0·489, -0·351) | |
| **Calibration slope^c^** | 0·862 (0·773, 0·951) | | 0·911 (0·838, 0·985) | |
| **Deviance^d^** | 68·5 | | 179·3 | |
| ^a^Adjusted using the Kaplan-Meier estimator to account for participants with follow-up duration < 10 years  ^b^Calibration-in-the-large >0 indicate underestimation of risk, calibration-in-the-large <0 indicate overestimation of risk. Numbers in parentheses are the 95% confidence intervals of the estimate.  ^c^Calibration slope ≠ 1 indicate poor spread of calibration. Numbers in parentheses are the 95% confidence intervals of the estimate.  ^d^Deviance values > 9·5 indicates poor goodness-of-fit at a significance level of 5%. | | | | |

## **Supplementary Table 8. Calibration of the recalibrated Pooled Cohort Equations for African Americans (PCE-AA) in the MEC1 (N = 6,900) and SEED cohort (N = 8,385).**

|  | **MEC1** | | **SEED** | |
| --- | --- | --- | --- | --- |
|  | **Male** | **Female** | **Male** | **Female** |
|  | **Chinese** | | | |
| **N observed events^a^** | 99·4 | 42·3 | 125·4 | 50·4 |
| **N predicted events** | 106·6 | 49·3 | 171·6 | 73·4 |
| **Calibration-in-the-large^b^** | -0·077 (-0·284, 0·117) | -0·174 (-0·497, 0·117) | -0·373 (-0·567, -0·191) | -0·410 (-0·717, -0·132) |
| **Calibration slope^c^** | 0·803 (0·573, 1·031) | 0·798 (0·525, 1·092) | 1·104 (0·853, 1·358) | 0·993 (0·634, 1·387) |
| **Deviance^d^** | 17·0 | 6·0 | 26·4 | 11·0 |
|  | **Malay** | | | |
| **N observed events^a^** | 92·3 | 49·1 | 232·9 | 151·2 |
| **N predicted events** | 88·0 | 47·1 | 254·0 | 123·4 |
| **Calibration-in-the-large^b^** | 0·037 (-0·177, 0·237) | 0·019 (-0·278, 0·289) | -0·157 (-0·294, -0·027) | 0·172 (0·001, 0·333) |
| **Calibration slope^c^** | 1·270 (1·024, 1·516) | 1·078 (0·844, 1·324) | 0·970 (0·802, 1·140) | 1·259 (1·075, 1·453) |
| **Deviance^d^** | 11·8 | 5·7 | 11·9 | 15·3 |
|  | **Indian** | | | |
| **N observed events^a^** | 103·8 | 63·3 | 207·6 | 110·3 |
| **N predicted events** | 99·8 | 63·6 | 210·3 | 125·3 |
| **Calibration-in-the-large^b^** | 0·005 (-0·197, 0·195) | -0·033 (-0·292, 0·206) | -0·057 (-0·199, 0·079) | -0·172 (-0·368, 0·013) |
| **Calibration slope^c^** | 1·208 (0·961, 1·452) | 0·752 (0·545, 0·968) | 1·164 (0·985, 1·343) | 1·029 (0·825, 1·243) |
| **Deviance^d^** | 5·9 | 7·2 | 5·4 | 6·5 |
|  | **Overall cohort** | | | |
| **N observed events^a^** | 447·5 | | 872·1 | |
| **N predicted events** | 454·6 | | 957·9 | |
| **Calibration-in-the-large^b^** | -0·029 (-0·124, 0·063) | | -0·136 (-0·205, -0·068) | |
| **Calibration slope^c^** | 0·965 (0·874, 1·057) | | 1·102 (1·026, 1·178) | |
| **Deviance^d^** | 16·2 | | 23·7 | |
| ^a^Adjusted using the Kaplan-Meier estimator to account for participants with follow-up duration < 10 years  ^b^Calibration-in-the-large >0 indicate underestimation of risk, calibration-in-the-large <0 indicate overestimation of risk. Numbers in parentheses are the 95% confidence intervals of the estimate.  ^c^Calibration slope ≠ 1 indicate poor spread of calibration. Numbers in parentheses are the 95% confidence intervals of the estimate.  ^d^Deviance values > 9·5 indicates poor goodness-of-fit at a significance level of 5%. | | | | |

## **Supplementary Table 9. Calibration of the SCORE2 Asia-Pacific model in the MEC1 (N = 5,447) and SEED cohort (N = 5,359).**

|  | **MEC1** | | **SEED** | |
| --- | --- | --- | --- | --- |
|  | **Male** | **Female** | **Male** | **Female** |
|  | **Chinese** | | | |
| **N observed events^a^** | 53·3 | 28·6 | 41·5 | 15·3 |
| **N predicted events** | 90·7 | 52·6 | 95·8 | 48·4 |
| **Calibration-in-the-large^b^** | -0·570 (-0·854, -0·310) | -0·636 (-1·030, -0·287) | -0·876 (-1·198, -0·584) | -1·185 (-1·738, -0·719) |
| **Calibration slope^c^** | 1·544 (1·174, 1·930) | 1·930 (1·528, 2·362) | 2·167 (1·619, 2·750) | 1·609 (0·997, 2·284) |
| **Deviance^d^** | 40·8 | 34·8 | 65·8 | 41·3 |
|  | **Malay** | | | |
| **N observed events^a^** | 49·6 | 20·3 | 93·4 | 39·1 |
| **N predicted events** | 45·3 | 29·2 | 79·3 | 38·5 |
| **Calibration-in-the-large^b^** | 0·079 (-0·214, 0·347) | -0·386 (-0·859, 0·022) | 0·144 (-0·069, 0·342) | -0·013 (-0·348, 0·289) |
| **Calibration slope^c^** | 1·730 (1·366, 2·103) | 1·599 (1·128, 2·089) | 1·398 (1·126, 1·677) | 1·477 (1·121, 1·863) |
| **Deviance^d^** | 17·0 | 16·4 | 10·9 | 10·8 |
|  | **Indian** | | | |
| **N observed events^a^** | 49·3 | 26·2 | 84·9 | 30·4 |
| **N predicted events** | 38·4 | 27·1 | 76·2 | 36·0 |
| **Calibration-in-the-large^b^** | 0·230 (-0·067, 0·500) | -0·044 (-0·455, 0·317) | 0·095 (-0·127, 0·302) | -0·188 (-0·569, 0·149) |
| **Calibration slope^c^** | 1·315 (0·940, 1·703) | 1·334 (0·928, 1·749) | 1·138 (0·818, 1·462) | 1·439 (1·019, 1·882) |
| **Deviance^d^** | 9·8 | 6·6 | 7·0 | 4·3 |
|  | **Overall cohort** | | | |
| **N observed events^a^** | 227·0 | | 303·8 | |
| **N predicted events** | 282·8 | | 374·1 | |
| **Calibration-in-the-large^b^** | -0·245 (-0·379, -0·116) | | -0·233 (-0·348, -0·122) | |
| **Calibration slope^c^** | 1·591 (1·432, 1·754) | | 1·495 (1·341, 1·652) | |
| **Deviance^d^** | 63·0 | | 56·3 | |
| ^a^Adjusted using the Kaplan-Meier estimator to account for participants with follow-up duration < 10 years  ^b^Calibration-in-the-large >0 indicate underestimation of risk, calibration-in-the-large <0 indicate overestimation of risk. Numbers in parentheses are the 95% confidence intervals of the estimate.  ^c^Calibration slope ≠ 1 indicate poor spread of calibration. Numbers in parentheses are the 95% confidence intervals of the estimate.  ^d^Deviance values > 9·5 indicates poor goodness-of-fit at a significance level of 5%. | | | | |

# **Supplementary Figures**


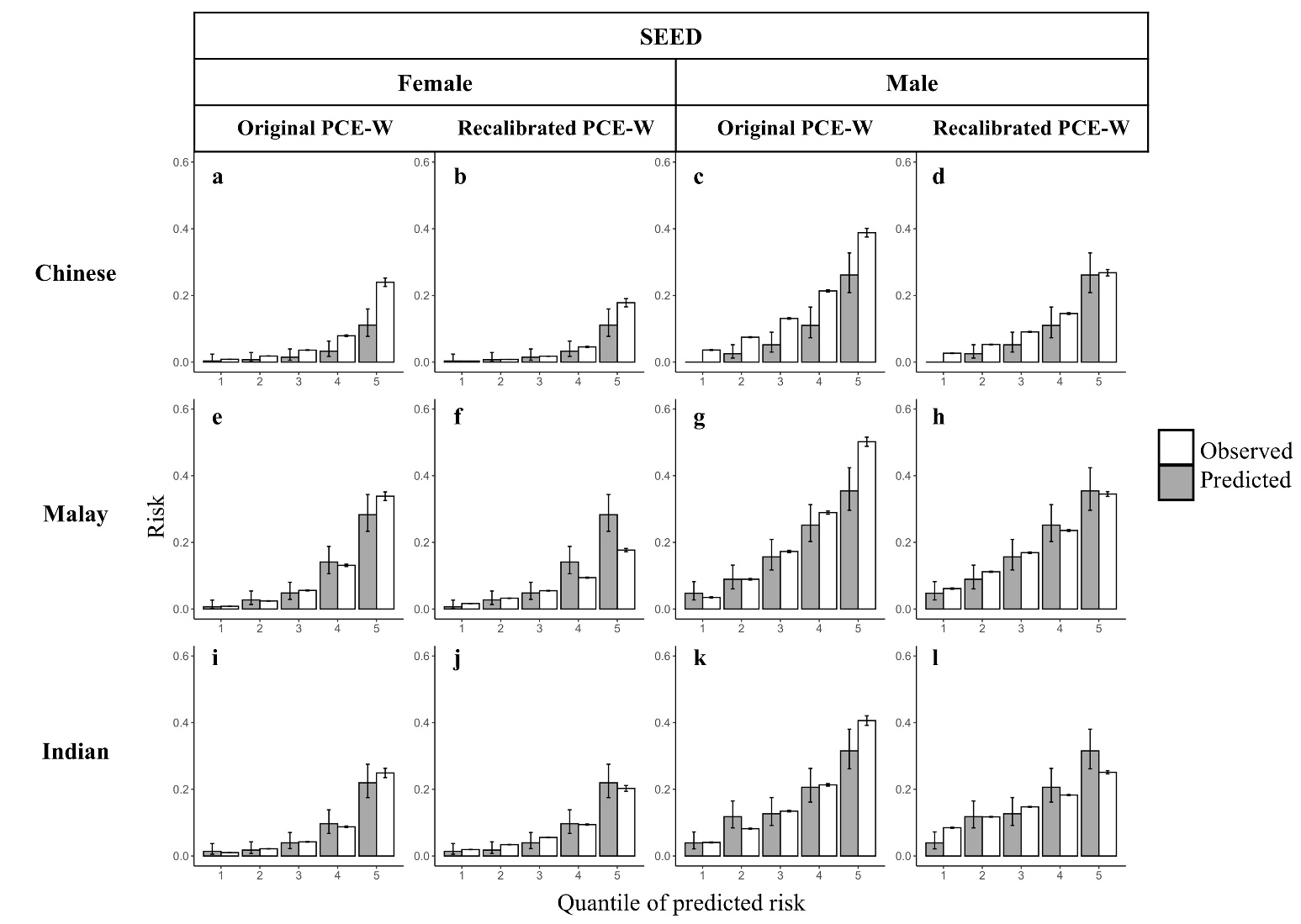


**Supplementary Figure 1. Comparison of the original Pooled Cohort Equations for Whites (PCE-W) versus recalibrated PCE-W in the Singapore Epidemiology of Eye Diseases study (SEED).** Quintiles of observed (grey) and predicted (white) risk are presented by sex (columns) and ethnicity (rows). The first and third columns (a, c, e, g, i, k) compare observed and predicted risk in the original PCE-W prediction model. The second and fourth columns (b, d, f, h, j ,l) compare observed and predicted risk in the recalibrated PCE-W prediction model. Error bars are 95% confidence intervals of the estimate.


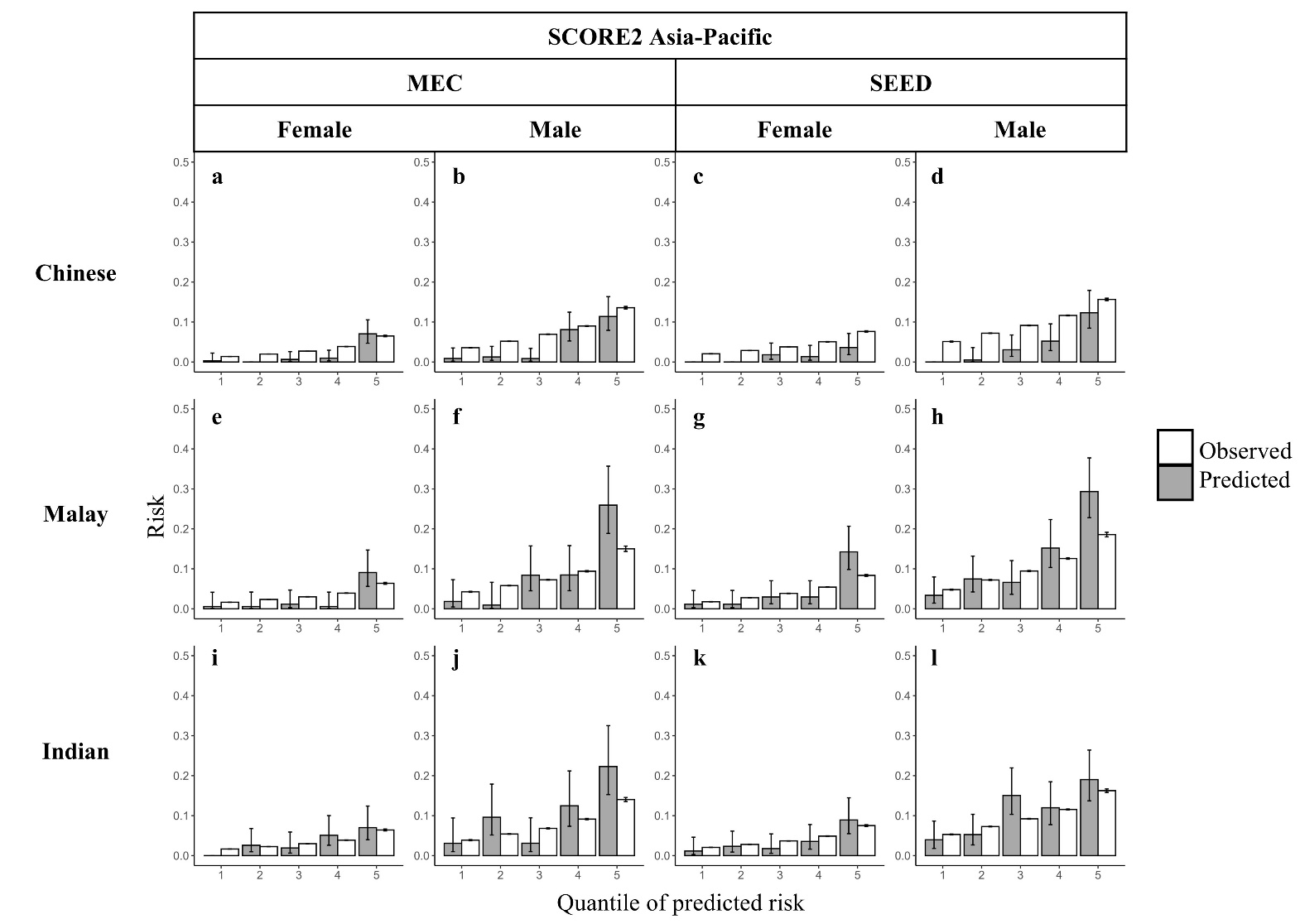


**Supplementary Figure 2. Comparison of the observed (grey) versus predicted (white) risks by the SCORE2 Asia-Pacific model (SCORE2-AP) in the Singapore Multi-Ethnic Cohort Phase 1 (MEC1) and Singapore Epidemiology of Eye Diseases study (SEED).** Quintiles of observed (grey) and predicted (white) risk are presented by sex (columns) and ethnicity (rows). The first two columns (a, b, e, f, i, j) present observed and predicted risk for MEC1. The third and fourth columns (c, d, g, h, k, l) present observed and predicted risk in SEED. Error bars are 95% confidence intervals of the estimate.


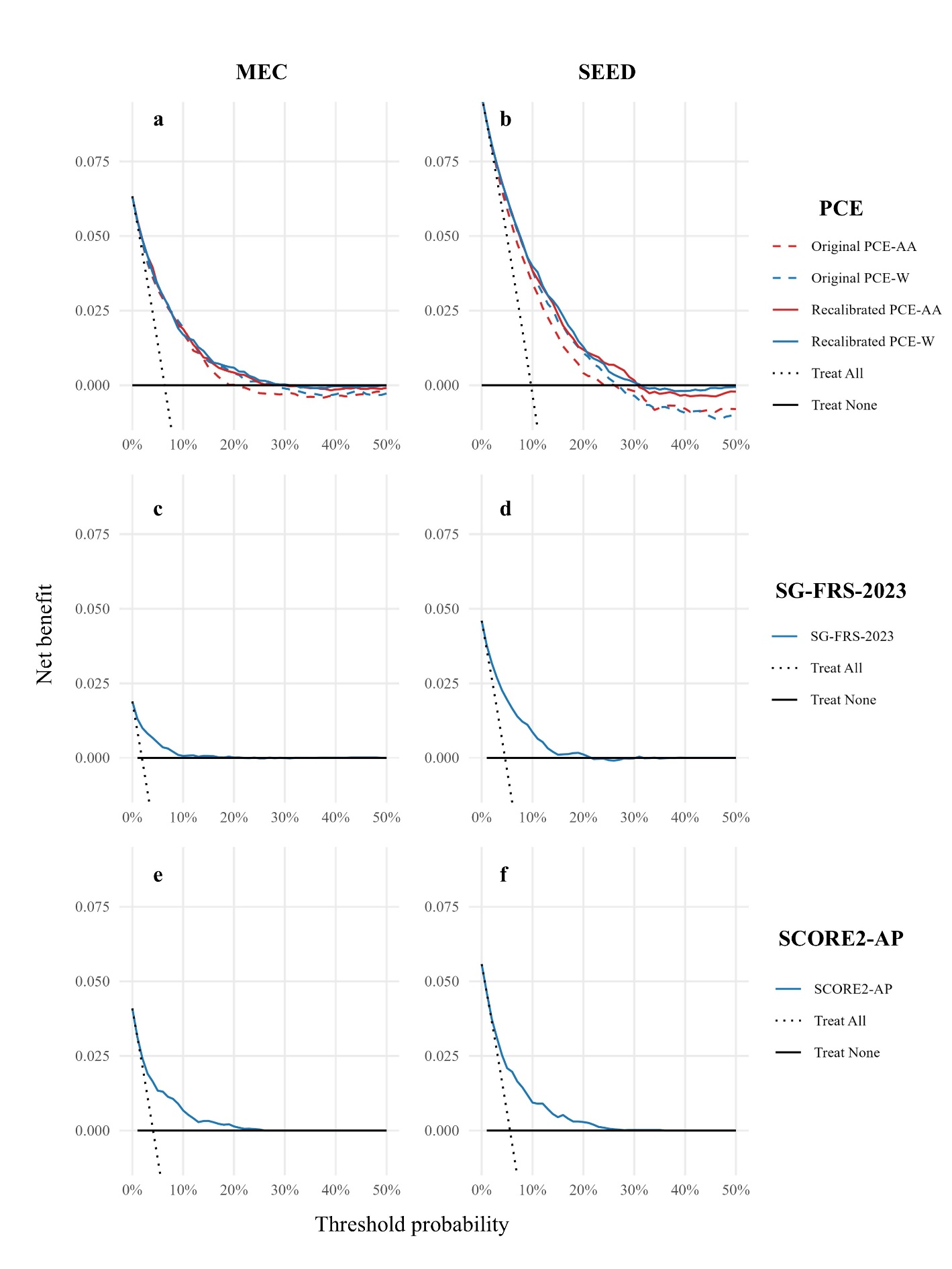


**Supplementary Figure 3. Decision curves showing net benefit across a range of threshold probabilities for CVD prediction using the Pooled Cohort Equations for Whites (PCE-W) and African Americans (PCE-AA), Singapore-modified Framingham Risk Score (SG-FRS-2023), and SCORE2 Asia-Pacific model (SCORE2-AP) in the Singapore Multi-Ethnic Cohort Phase 1 (MEC1) and Singapore Epidemiology of Eye Diseases study (SEED).**
